# Profound perturbation induced by triclosan exposure in mouse gut microbiome: a less resilient microbial community with elevated antibiotic and metal resistomes

**DOI:** 10.1186/s40360-017-0150-9

**Published:** 2017-06-12

**Authors:** Bei Gao, Pengcheng Tu, Xiaoming Bian, Liang Chi, Hongyu Ru, Kun Lu

**Affiliations:** 1 Department of Environmental Sciences and Engineering, University of North Carolina at Chapel Hill, Chapel Hill, NC 27599 USA; 2 Department of Environmental Health Science, University of Georgia, Athens, GA 30602 USA; 3 Department of Population Health and Pathobiology, North Carolina State University, Raleigh, 27606 USA

**Keywords:** Triclosan, Gut microbiome, Antibiotic resistance, Metal resistance, Resistome, Triclosan resistance

## Abstract

**Background:**

Environmental chemical-induced perturbations of gut microbiome are associated with a series of adverse health outcomes. The effects of triclosan on human health have been controversial in recent years. The purpose of this study is to investigate the functional impact of triclosan on the mouse gut microbiome and the link between triclosan exposure and resistomes in gut bacteria.

**Methods:**

We combined 16S rRNA gene sequencing and shotgun metagenomics sequencing to examine the compositional and functional impact of triclosan exposure on the gut microbiota of C57BL/6 mice.

**Results:**

16S rRNA sequencing results revealed that 13-week triclosan exposure in drinking water induced significant perturbations in mouse gut bacterial assemblages with distinct trajectories compared to controls. Metagenomics sequencing results indicated a remarkable enrichment of gut bacterial genes related to triclosan resistance, stress response, antibiotic resistance and heavy metal resistance.

**Conclusions:**

Triclosan exposure has a profound impact on the mouse gut microbiome by inducing perturbations at both compositional and functional levels. To our best knowledge, this is the first evidence regarding the functional alterations of gut microbiome induced by triclosan exposure, which may provide novel mechanistic insights into triclosan exposure and associated diseases.

## Background

The host and its gut-residing commensal microbiota usually have a mutually beneficial relationship, which confers health benefits for the host resulting from their frequent metabolic interactions [1]. For example, a properly-functioning immune system from birth is inseparable from the communications with gut bacteria [2]. However, this relationship can be readily affected by various endogenous (e.g. genetics) and exogenous factors (e.g. diet) [3]. For instance, it is well-established that the composition of gut microbiota can be changed by both long-term and short-term dietary consumptions in human [4, 5]. In addition, recent studies demonstrated that exposure to environmental agents such as heavy metals, pesticides and antibiotics is able to induce the perturbations of gut microbial community, and alterations in terms of gut microbiome-related metabolic functions occur consequently [6–8]. Accumulating evidence indicates that a variety of adverse health outcomes ensue as a result of perturbed gut microbiome, such as inflammatory bowel disease, obesity, liver diseases and colon cancer [2]. Thus, a better understanding of the underexplored relationship between environmental agents and the microbiome perturbations with its resultant health implications is imperative and noteworthy.

Triclosan [5-chloro-2-(2,4-dichlorophenoxy) phenol] has been used worldwide for more than 40 years in many areas, such as personal care products and clinical settings [9]. Triclosan is also an emerging environmental contaminant as its detected concentration is up to 2.3 μg/L in U.S. surface waters [10]. A study conducted in 2008 revealed that nearly three quarters of the human urine samples were detected to have triclosan with the concentrations ranging up to 3.79 ppm in U.S. [11]. Triclosan exposure is prevalent in pregnant women and children, and interestingly, is correlated with higher socioeconomic status [12, 13]. A number of diseases especially liver malfunction and carcinogenesis have been linked to triclosan exposure by multiple mechanisms, including oxidative stress, epigenetic factors, inflammation and cell proliferation and fibrogenesis [9]. Intriguingly, most recent studies observed that triclosan exposure was able to induce gut microbiome perturbations in animal models. For example, it has been shown that triclosan shifted the gut microbiome structure of adult zebrafish in one week [14]; likewise, triclosan exposure also changed the gut microbial composition in fathead minnow [15]. In addition, another study revealed that the gut microbiota in adolescent rats was more vulnerable than that in adult rats, indicating a specific window of susceptibility of gut microbiome perturbations [16]. Given the crucial role that the gut microbiome plays in human health coupled with the capacity of triclosan to trigger gut microbiome alterations, it is of significance to elucidate the mechanistic basis for triclosan-induced diseases through the lens of the gut microbiome. Knowing the compositional change alone does not necessarily lead to a better understanding of the functional alterations and its associated health prospects because the gut bacterial functions vary dramatically with distinct metabolic activities under different circumstances [17]. However, the impact of triclosan exposure on gut microbiome at functional level still remains largely elusive. Therefore, it is in need to probe the functional alterations in triclosan-perturbed microbiome. In addition, it is reported that triclosan concentration is correlated with the proportion of triclosan-resistant bacteria within cultivable benthic bacterial community, suggesting that triclosan exposure might promote the enrichment of bacteria that possess resistance capacity [18]. Although controversy exists regarding a causal relationship between antibiotic resistance and triclosan exposure, considering the cross-resistance due to shared resistance mechanisms and the growing threat that antibiotic resistance poses, there is a necessity to examine triclosan resistance as well as antibiotics resistome in the gut microbiome with triclosan exposure.

In the present study, we combined 16S rRNA gene sequencing and shotgun metagenomics sequencing for the investigation of the effects of triclosan exposure on mouse gut microbiome and its metabolic functions. Our 16S rRNA sequencing result revealed pronounced difference in bacterial assemblages with distinct trajectories. The metagenomics sequencing results showed significant alterations of gut bacterial gene repertoires, with enrichment of genes that indicated triclosan resistance. In addition, we found remarkable enrichment of genes involved in stress response, antibiotic resistance and heavy metal resistance. To our best knowledge, this is the first evidence regarding the functional alteration of the gut microbiome induced by triclosan exposure, these findings may introduce a new mechanism, that is, triclosan exposure contributes to human diseases by perturbing the gut microbiome.

## Methods

### Animals and exposure

The animal experiment protocol was approved by the University of Georgia Institutional Animal Care and Use Committee. A total of 10 C57BL/6 mice (8 weeks old), purchased from Jackson Laboratories were housing in the animal facility of University of Georgia for one week for acclimation before triclosan treatment (standard pelleted rodent diet and tap water ad libitum provided). After the acclimation, the mice were then randomly assigned to either a control or treatment group. 2 ppm triclosan water solution (the concentration used in the present study is 100 times lower than that can promote liver carcinogenesis) was provided for the treatment group replacing tap water when starting exposure [19]. They were housed under the environmental conditions as 22 °C, 40–70% humidity, and a 12:12 hr light:dark cycle and were provided with standard pelleted rodent diet and water (control group) or triclosan solution (treatment group) ad libitum over the course of 13 weeks. Fecal samples for 4-week and 13-week exposure were collected from individual mouse and kept at −80 °C immediately for further analysis.

### 16S rRNA gene sequencing

16S rRNA gene sequencing was performed as described in Lu et al. (2014) [6]. We isolated DNA from fecal pellets using PowerSoil® DNA isolation kit according to the manufacturer’s instruction. And then DNA was amplified using 515 F and 806R primers [20] targeting the V4 regions of 16S rRNA of bacteria, followed by normalization, barcoding procedure and finally pooled for the construction of the sequencing library. The resultant DNA was quantified using Qubit 2.0 Fluorometer, and then sequenced using Illumina MiSeq (500 cycles v2 kit) in the Georgia Genomics Facility of University of Georgia. Paired-reads were assembled using Geneious software (Biomatters, Auckland, New Zealand), and operational taxonomic unit (OTU) picking and diversity analysis were conducted using Quantitative Insights into Microbial Ecology (QIIME) software.

### Shotgun metagenomics sequencing

Shotgun metagenomics sequencing was performed as described in Gao, Bei, et al. (2016) [7]. DNA (10 ng/μL) from individual mouse was fragmented using the Bioruptor UCD-300 sonication device, and then the library was constructed using the Kapa Hyper Prep Kit according to the manufacturer’s instruction. The resultant DNA was quantified using Qubit 2.0 Fluorometer, and sequenced using an Illumina NextSeq High Output Flow Cell in the Georgia Genomics Facility of University of Georgia. Raw fastq data were uploaded into the MG-RAST metagenomics analysis server (version 3.6) for comparative metagenomic analysis. The SEED Subsystem database was applied as the annotation source. (parameters: Max. e-Value Cutoff 1e-5, Min. Identity Cutoff 60%, and Min. Alignment Length Cutoff of 15) [21].

### Statistical analysis of data

The compositional difference among individual gut microbiome was assessed by a nonparametric test via Metastats software (http://metastats.cbcb.umd.edu/) as described previously [22]. Alpha rarefaction was performed using observed OTUs metrics via Quantitative Insights into Microbial Ecology (QIIME) software. Also, the Jackknifed beta diversity and hierarchical clustering analysis via Unweighted Pair Group Method with Arithmetic Mean (UPGMA) was used for sample clustering. Moreover, principle coordinate analysis (PCoA) was applied to examine the difference of beta diversity based on the UniFrac distance metric [23]. In addition, heat maps were generated using a hierarchical clustering algorithm to visualize the comparison of gene abundance. The abundance of bacterial genes was compared with a Student’s t test, and the result was considered to be significant if *p* < 0.05.

## Results

### Triclosan exposure significantly changed the gut microbiome composition

**Fig. 1:**
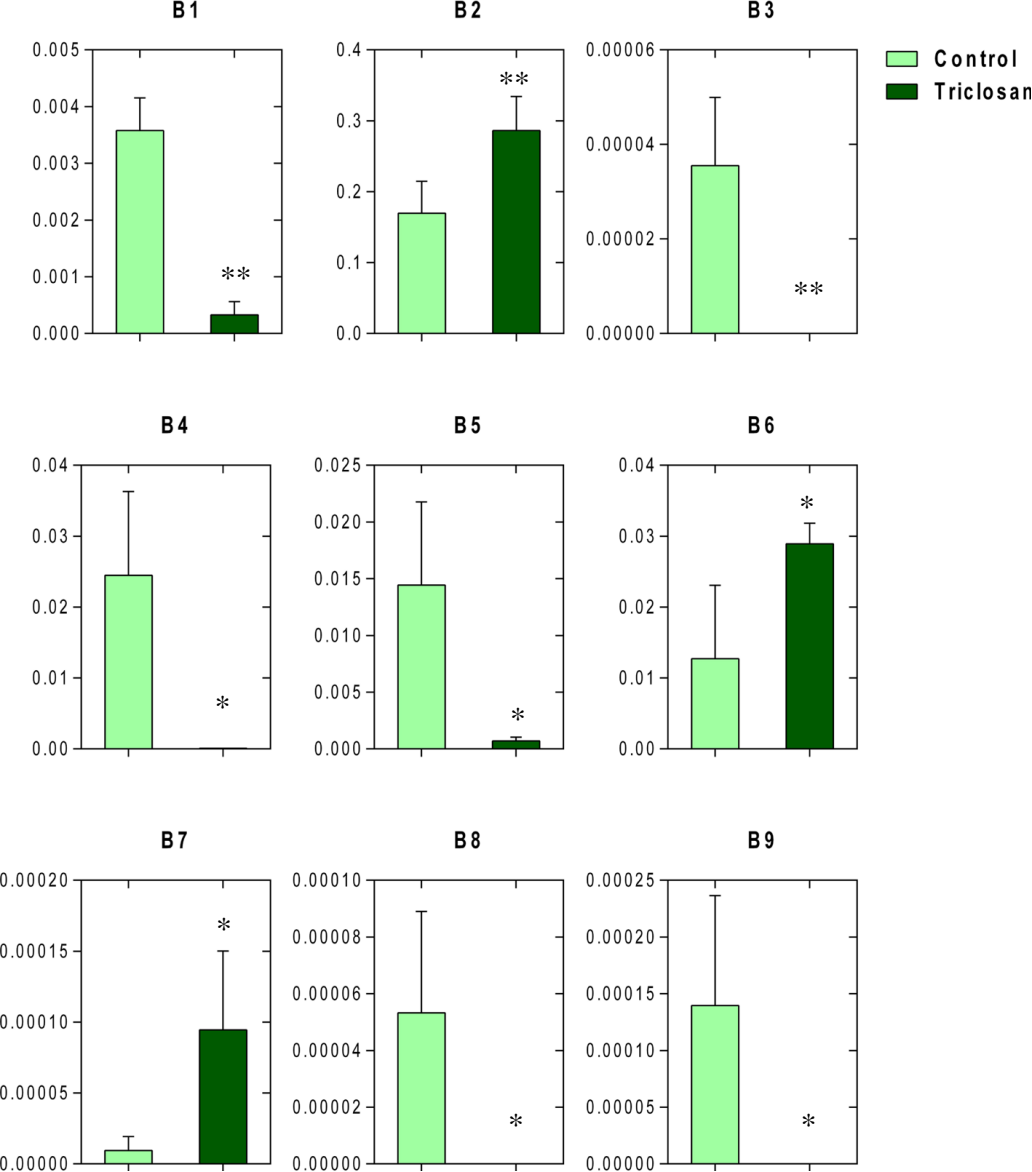
Significantly-altered bacteria at the family level in the gut microbial community by comparison between the triclosan and control groups, with the y axis for relative abundance. (* *p* < 0.05; ** *P* < 0.01) [Abbreviations: c, class; o, order; f, family. B1 (*c_Mollicutes;o_RF39;f_Unassigned*); B2 (*c_Clostridia;o_Clostridiales;f_Unassigned*); B3 (*c_Clostridia;o_Clostridiales;Other*); B4 (*c_Bacilli;o_Turicibacterales;f_Turicibacteraceae*); B5 (*c_Clostridia;o_Clostridiales;f_Clostridiaceae*); B6 (*c_Bacilli;o_Lactobacillales;f_Lactobacillaceae*); B7 (*c_Bacilli;o_Lactobacillales;f_Streptococcaceae*); B8 (*c_Clostridia;o_Clostridiales;f_Christensenellaceae*); B9 (*c_Bacilli;Other;Other*)]

To investigate the triclosan-induced change of the composition in mouse gut microbiome, we compared the abundance of gut bacteria between the treatment and control groups after 13-week triclosan exposure. 13-week exposure substantially altered the microbial community manifested by a series of shifted bacterial families. Figure 1 shows the identified gut bacteria from 16S rRNA sequencing reads at the family level with significant difference between triclosan and control groups. The abundance of 9 gut bacterial families was altered by triclosan exposure, with 6 decreased and 3 increased ones. Of these, B3 (*c_Clostridia;o_Clostridiales;Other*), B4 (*c_Bacilli;o_Turicibacterales;f_Turicibacteraceae*), B8 (*c_Clostridia;o_Clostridiales;f_Christensenellaceae*) and B9 (*c_Bacilli;Other;Other*) were completed depleted by triclosan.

### Triclosan-induced diversity alteration and trajectory remodeling

**Fig. 2:**
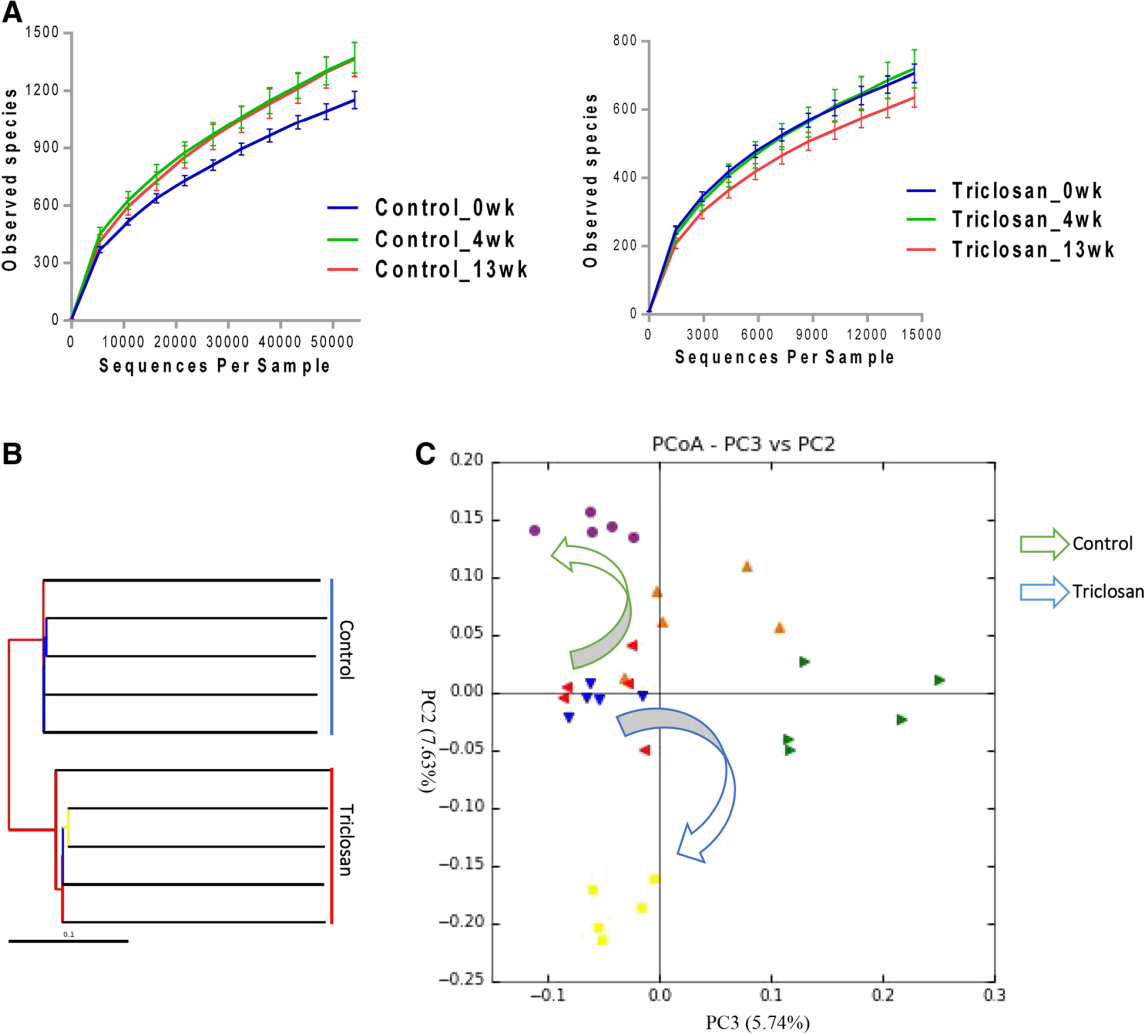
**a** The alpha rarefaction at 0 week, 4 week, 13 week in control and triclosan groups, respectively. **b** Hierarchical clustering analysis by UPGMA with the UPGMA distance tree constructed at a distance of 0.1. **c** The trajectories of gut microbiota in the control and triclosan group by principle coordinate analysis with arrows depicting the developmental trend

Alpha and beta diversities refer to the biodiversity within a group of samples or between each pair of groups, respectively [24]. We first measured differences of alpha diversity in the overall bacterial community over the 13-week course in either the control or treatment group using the observed OTUs metric. Figure 2a shows the alpha rarefaction at different time points in the control and treatment groups, respectively. As showed, in the control group, the alpha diversity increased in the first 4 weeks, and then remained approximately unchanged till the end of the experiment. In comparison, the alpha diversity of treatment group remained unchanged for the first 4 weeks and then decreased with the exposure going on for another 9 weeks, which is consistent with previous study that antibiotics are able to reduce species richness in gut microbial community [8]. A lower alpha diversity represents a more vulnerable microbial community for the reason that species richness is essential for conferring resilience in ecosystems [17]. We next compared the degree of dissimilarity between the treatment and control groups using the jackknifed beta diversity and hierarchical clustering analysis via the unweighted pair group method with arithmetic mean (UPGMA) (Fig. 2b), which shows that the individuals from the two groups clustered together in their own groups and the treatment and control groups were well separated from each other, indicating distinct microbial structures. In addition, the PCoA plot (Fig. 2c) shows the beta diversity distance matrix of the mouse gut microbiota in the treatment and control groups at 0, 4 and 13 weeks, which portrayed distinct changing trajectories with triclosan treatment. Different alterations of alpha diversity across time between the two groups also supported the notion that a distinct changing pattern was triggered by triclosan exposure.

### Genetic alterations in mouse gut microbiome related to triclosan resistance

**Fig. 3:**
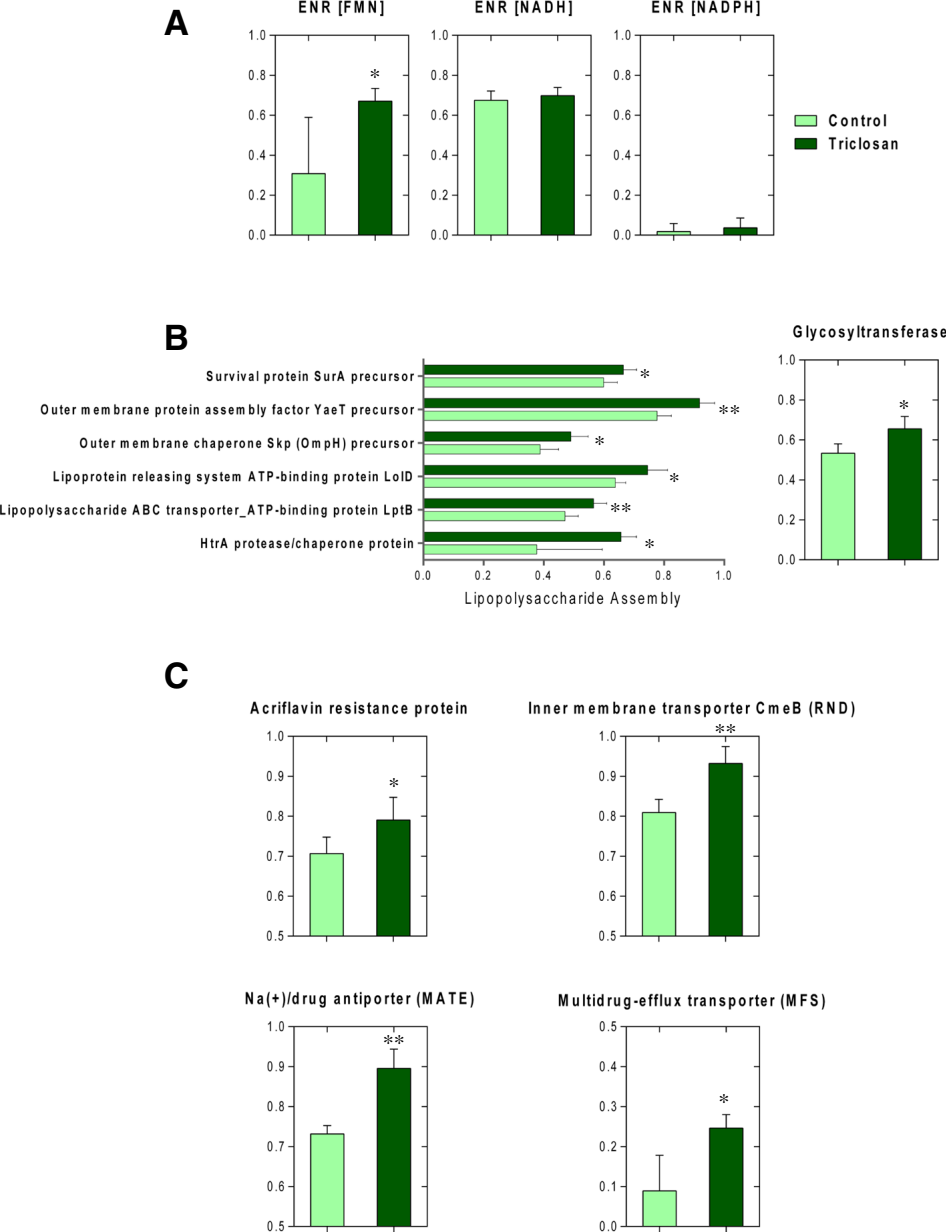
Triclosan exposure altered the relative abundances of gut bacterial genes encoding ENRs in bacteria (**a**); LPS biosynthesis and assembly (**b**); efflux pumps (**c**). (* *p* < 0.05; ** *P* < 0.01)

The comparison of the mouse gut bacterial metagenome showed enrichment of bacterial genes suggesting potential triclosan resistance. Enoyl-[acyl carrier protein] reductase (ENR) is the target site of triclosan in most susceptible microorganisms [25]. Overproduction of FabI (a common ENR found in bacteria) or expression of alternate enzymes usually can lead to triclosan resistance [26]. We compared several bacterial genes encoding ENR enzymes. The relative abundance of ENR (FMN) was significantly enriched in the treatment group (Fig. 3a). However, ENR (NADH) and ENR (NADPH) showed no significant difference. Moreover, lipopolysaccharide (LPS) in the membrane of gram-negative bacteria, especially the core oligosaccharides confers intrinsic resistance for bacteria by creating a physical barrier against unfavorable hydrophobic agents [27]. We then compared the relative abundance of the bacterial genes involved in the LPS formation. The bacterial gene encoding glycosyltransferase, which is involved in the biosynthesis of the core oligosaccharides, and multiple genes related to LPS assembly pathway (Fig. 3b) showed significant enrichment in the triclosan group. Therefore, it is possible that the gut bacteria obtained potential resistance to triclosan resulting from the change of the membrane structure. In addition, multiple bacterial genes associated with multidrug resistance efflux pumps have increased abundances after triclosan exposure, including genes encoding acriflavin resistance protein, Na(+)/drug antiporter, multidrug-efflux transporter and inner membrane transporter CmeB (Fig. 3c). Multidrug resistance efflux pumps can be involved in triclosan resistance as well as resistance to a series of antibiotics.

### Enrichment of bacterial genes involved in stress response after triclosan exposure

**Fig. 4:**
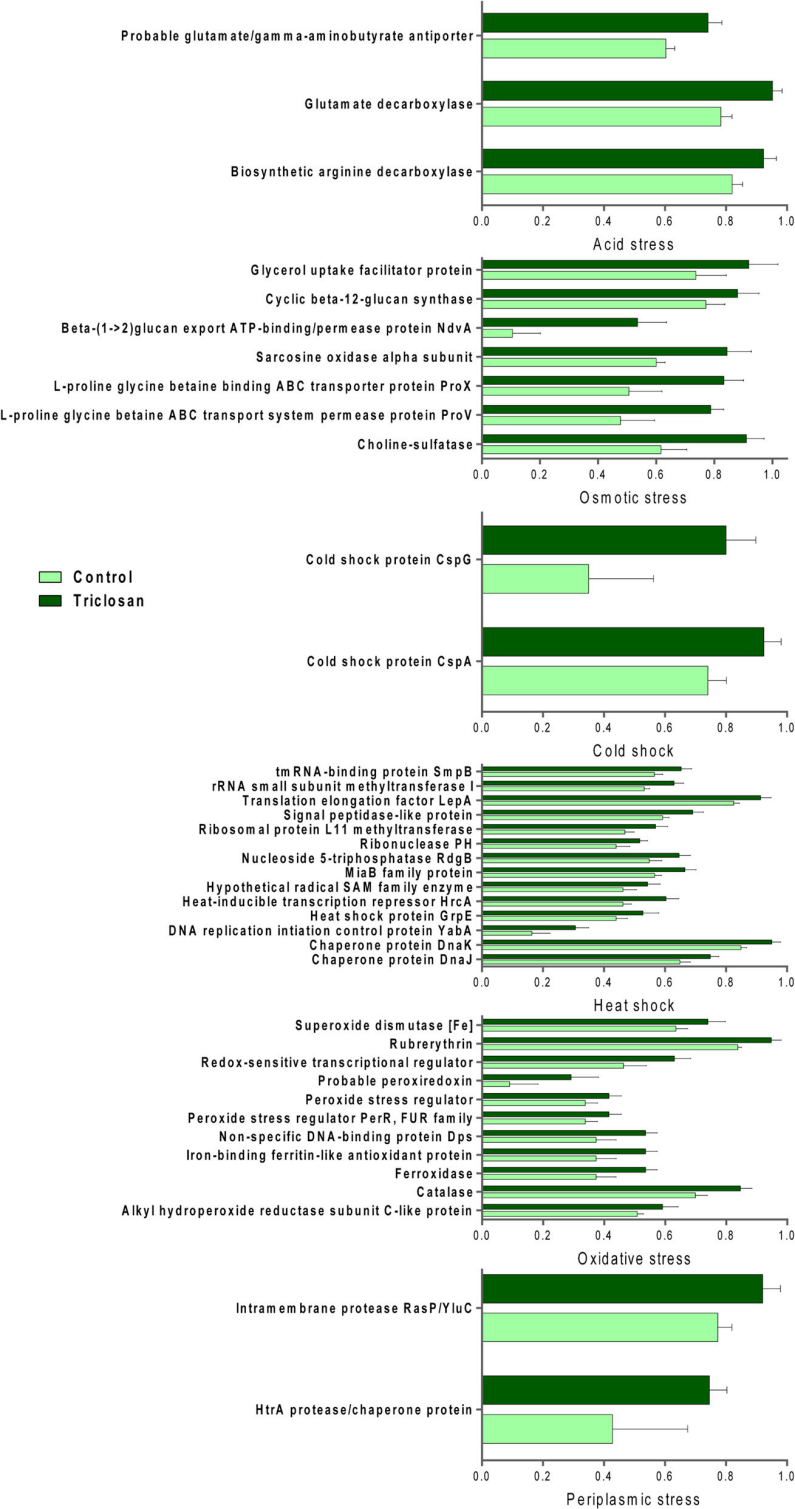
Triclosan exposure perturbed the relative abundances of gut bacterial genes involved in stress response. (all comparisons listed here are significant, *p* < 0.05)

Stress response-related functions in bacteria help facilitate the transition under environmental fluctuations, hence increasing the chances of survival [28]. By comparative metagenomic analysis, bacterial genes involved in stress response exhibited significant enrichment after triclosan exposure. Figure 4 shows the distribution of bacterial genes involved in bacterial stress response at the functional level with the SEED subsystem as the annotation source. As we can see, genes involved in acid stress, cold shock, heat shock, osmotic stress, oxidative stress and periplasmic stress were significantly enriched (*p* < 0.05). Enhanced bacterial response to stress might indicate intensified stress exerted by triclosan in the gut milieu.

### Triclosan exposure raised the antibiotic and metal resistomes in the gut microbiome

**Fig. 5:**
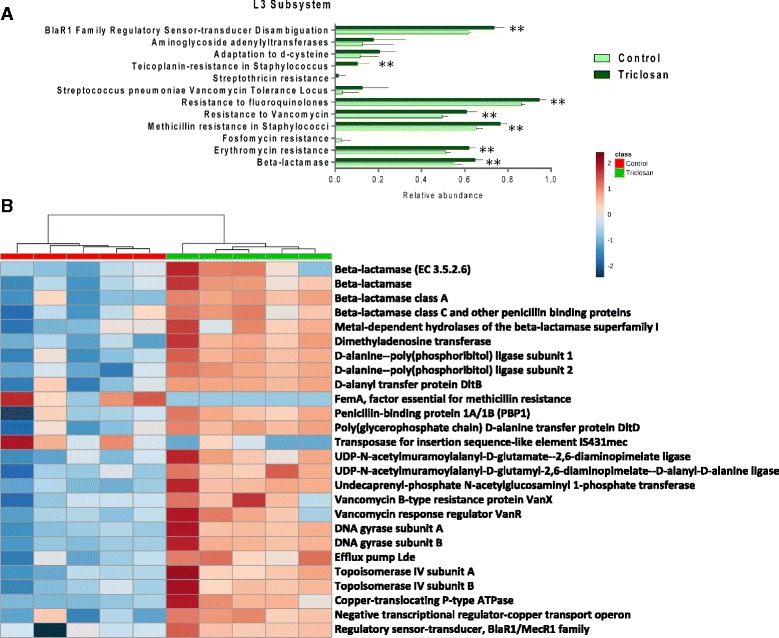
**a** Comparative metagenomic analysis in terms of antibiotic resistance at the Level 3 subsystem. (* *p* < 0.05; ** *P* < 0.01) **b** Heat map constructed using the relative abundances of antibiotic resistance-related bacterial genes that are significantly different between the triclosan and control group. (*p* < 0.05)

**Fig. 6:**
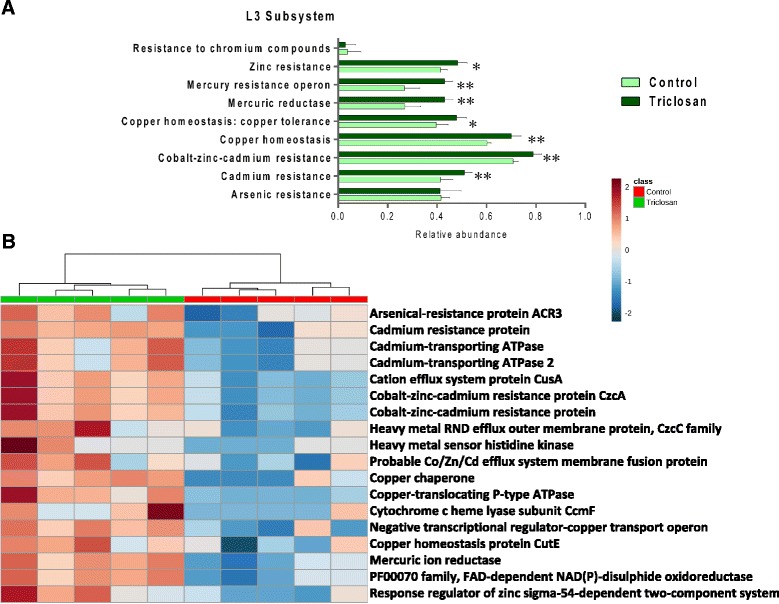
(**a**) Comparative metagenomic analysis in terms of metal resistance at the Level 3 subsystem. (* *p* < 0.05; ** *P* < 0.01) (**b**) Heat map constructed using relative abundances of metal resistance-related bacterial genes that are significantly different between the triclosan and control group. (*p* < 0.05)

Metagenomic comparison revealed a striking enrichment in the antibiotic and metal resistomes of the mouse gut microbiota after triclosan treatment. Figure 5a showed the comparison in antibiotic resistance at Level 3 with the SEED subsystem as the annotation source. Of these, bacterial functions (Level 3) subsystems including beta-lactamase, erythromycin resistance, methicillin resistance in Staphylococci, resistance to vancomycin, resistance to fluoroquinolones, teicoplanin resistance in Staphylococcus, BlaR1 family regulatory sensor-transducer disambiguation have significantly increased compared to controls. Such an increase in antibiotic resistome in the gut microbiome can be a threat for the effectiveness in treating infections. Likewise, Fig. 6a revealed the comparison of genes involved in heavy metal resistance in gut bacteria. Bacterial functions (Level 3) including cadmium resistance, cobalt-zinc-cadmium resistance, copper homeostasis, copper tolerance, mercuric reductase, mercury resistance operon, zinc resistance showed significant enrichment compared to controls. In addition, for the comparison at the functional level, heat maps (Figs. 5b, 6b) representing the distribution of genes involved in antibiotics resistance and heavy metal resistance display clear enrichment in abundance and a consistent clustering pattern. The co-occurrence of increased antibiotics and heavy metal resistomes supported the existence of the co-selection for antibiotic and heavy metal resistance [29].

## Discussion

Growing evidence has associated perturbed gut microbiome with a number of adverse health outcomes. A better understanding of the interplay between environmental exposure and the gut microbiome-related metabolic alterations will aid in delineating the mechanistic basis of diseases associated with dysbiosis and eventually minimizing susceptibility in human. Triclosan, a commonly-used antimicrobial chemical, has raised long-standing public concern due to the controversy that it might exert an adverse impact on human health and facilitate the spread of antibiotic resistance [30]. In the present study, we used high-throughput 16S rRNA gene sequencing and shotgun metagenomics sequencing to probe the compositional and functional alterations by triclosan exposure on the gut microbiome of C57BL/6 mice. The results clearly demonstrated that triclosan exposure changed the composition and diversity of the mouse gut microbiome and reprogrammed its developmental trajectory. More importantly, triclosan exposure induced significant enrichment of bacterial genes that might confer potential triclosan resistance in gut bacteria. In addition, bacterial genes involved in stress response, antibiotic resistance and heavy metal resistance were also remarkably enriched after triclosan exposure. These findings supported the notion that gut microbiome perturbations can be a novel mechanism by which environmental toxic chemicals adversely influence human health [6].

Time is one of the key factors that can affect the composition and diversity of gut microbiome. For example, the proportional representation of gut bacterial genes involved in folate biosynthesis decreased by aging, however, in the case of cobalamin it increased [31]. Likewise, the ratio of *Firmicutes* to *Bacteroidetes* fluctuates across the lifespan [32]. In the present study, the mouse gut microbiome evolved over time with or without exposure. However, the trajectories diverged, ending up with two totally different microbial communities with disparate metabolic functions in the triclosan and control groups. The effect of triclosan exposure on the trajectory suggests that triclosan alters the normal developmental path of the gut microbiome, which is consistent with a recent study that another environmental toxic chemical arsenic was also found to be able to alter the trajectory of mouse gut microbiome [33]. This exposure-shaped microbial community distinct from the one in the control group may lead to adverse health effects for different functional roles that it has in the gut-host interactions.

Resilience is the ability of an ecosystem to tolerate certain degrees of disturbance before diverging its trajectory to establish a new equilibrium [34]. The resilience in a given ecosystem is often associated with its diversity, that is, communities with higher species richness are less vulnerable to interruptions because of the higher utilization efficiency resulting from the specialization of various species for each potentially limiting source [35]. In the present study, triclosan reshaped the gut micro-ecosystem. As shown in Fig. 2c, the trajectories oriented similarly at first and then went towards opposite directions. Likewise, in the control group, the alpha diversity increased in the first 4 weeks and remained unchanged (Fig. 2a). By contrast, in the triclosan group, the alpha diversity remained unchanged at first and decreased to lower than the start point at the end of the treatment course (Fig. 2a). The two distinct patterns in alpha diversity transition can be explained in view of the resilient capacity of gut micro-ecosystem. Without exposure, the microbiota thrived leading to rocketing species richness, and soon the ecological saturation was reached therefore the alpha diversity being constant afterwards. However, in the case of the triclosan group, the resilience managed to maintain the integrity of the gut micro-ecosystem in the beginning of the exposure since the triclosan-induced perturbations could be still within the tolerance. However, with the exposure going on, the perturbations accumulated and eventually exceeded the degree that the ecosystem can endure. Thus, a vicious circle may be formed with waxing perturbations and waning resilience capacity, which results in the divergence of trajectory leading to the establishment of a new ecological equilibrium.

An enrichment of bacterial genes involved in potential triclosan resistance was induced in the mouse gut microbiome after triclosan exposure. Mechanisms underlying bacterial triclosan resistance include target site mutation or overexpression, membrane structure alterations and efflux pumps [26]. Foremost, we discovered a significant increase of the bacterial gene encoding ENR (FMN). Meanwhile, the relative abundance of genes encoding ENR (NADH) and ENR (NADPH) did not show significant difference. Triclosan can act on a specific target ENR, thereby interrupting bacterial lipid synthesis and performing its antimicrobial function [36]. It is demonstrated that mutation in fabI that encodes ENR in *Escherichia coli* is responsible for the triclosan resistance in mutants [37]. Complex ENR diversity exists among bacteria, which enables another potential mechanism for triclosan resistance: expression of alternative triclosan-insensitive enzymes [38]. The reaction that ENR catalyzes usually prefers either NADH or NADPH as a cofactor [38]. However, FabK, the ENR found in *Streptococcus pneumoniae*, uses FMN as the cofactor [39]. The enrichment of the gene encoding ENR (FMN) suggested enhanced triclosan resistance by expression of alternative enzymes under the selection of triclosan exposure.

In addition, we also discovered alterations of gut bacterial genes related to bacterial membrane structure, specifically, lipopolysaccharides (LPS) biosynthesis and assembly. The outer membrane of gram-negative bacteria is linked to another antibiotic resistance mechanism for providing a physical barrier against antibiotics or other antimicrobial compounds [27]. For example, it is reported that the exclusionary property of the outer membrane is attributed to the intrinsic resistance to low-level triclosan exposure in *Pseudomonas aeruginosa* [40]. Moreover, incomplete core oligosaccharide of LPS would result in high susceptibility to antibiotics in *Salmonella* [41]. The length and branches of core oligosaccharide is essential for the barrier against hydrophobic antibiotics. As a result, an enrichment of genes related to the biosynthesis of core oligosaccharides can be associated with enhanced triclosan resistance [27]. Thus, increased abundance of the gene encoding the key enzyme glycosyltransferase in the biosynthesis of core oligosaccharides, in concert with multiple genes involved in LPS assembly pathway, suggested increased triclosan resistance also resulting from a reinforced membrane barrier in gut bacteria.

Finally, the abundances of several bacterial genes related to multidrug-resistance efflux pumps significantly increased after triclosan exposure. It is well-recognized that efflux pumps are associated with triclosan resistance as well as antibiotics resistance [42]. For example, triclosan is the substrate for the AcrAB efflux pump that is responsible for the triclosan-resistant property in *E. coli* and *Salmonella enterica* [43, 44]. Likewise, TriABC associated with OpmH is a triclosan-specific efflux pump that confers triclosan resistance in *Pseudomonas aeruginosa* [45]. Triclosan is the substrate for most efflux pumps that belong to resistance nodulation cell division (RND) family [42]. Accordingly, overexpression of CmeB, a member of RND family, can be linked to decreased susceptibility of triclosan exposure [46]. Increased abundance of the gene encoding CmeB in *Campylobacter jejuni* was recognized in the gut bacteria of triclosan-treated mice, suggesting potential selection for bacteria that are equipped with such efflux pumps under triclosan exposure. Since the efflux pumps are usually associated with multidrug resistance, the enrichment of genes related to multidrug-resistance efflux pumps may simultaneously lead to a raised antibiotic resistome in gut mictobiota [42]. For instance, it is demonstrated in a previous study that overexpression of Cmeb confers resistance to multiple antibiotics such as ciprofloxacin, erythromycin, ampicillin, chloramphenicol and tetracycline [46].

Taken together, the enrichment of bacterial genes involved in triclosan resistance in mouse gut microbiome might suggest the existence of potential selection by triclosan exposure. In the beginning, a minority of bacteria that possessed triclosan resistance-related genes soon became in advantage under the environmental stress exerted by triclosan exposure, and before long, those bacteria would outcompeted other species with higher triclosan susceptibility. The overall community would gradually remodel towards a new equilibrium where triclosan-resistant members became dominant. During this transient progress, the species richness declined with concomitant weakening resilience resulting from the relative rareness of triclosan resistance-related genes in natural environment.

The accelerating spread of antibiotic resistance has become a serious public concern [47]. Triclosan resistance is closely associated with antibiotic resistance for the reason that they shared underlying mechanisms such as impermeable membrane barrier and efflux pumps [48]. Therefore, the enrichment of bacterial genes related to triclosan resistance may also confer resistance to a range of antibiotics. Besides, an enrichment of bacterial genes involved in multi-antibiotic resistance was identified in the gut microbiome after triclosan exposure. The connection between triclosan and antibiotic resistance still remains controversy, however in this study, an overrepresented antibiotic resistome was recognized. As an anthropogenic product, the use of triclosan may partially explain that the presence of antibiotic resistance genes was positively associated with the proximity of human activities [49]. More importantly, the triclosan contamination in both terrestrial and aquatic environments due to its massive usage and persistent quality would exert an obvious selection pressure for antibiotic resistance genes in natural environments, jeopardizing the potency of antibiotics in treating human pathogens that acquired resistance genes via horizontal gene transfer [50]. In addition, an enrichment of bacterial genes involved in heavy metal resistance has also been identified. Such co-occurrence supported the co-selection between antibiotic resistome and heavy metal resistome with three underlying mechanisms: co-resistance, cross-resistance and co-regulation [51].

Our findings demonstrated that triclosan exposure perturbed the mouse gut microbiome and its functional profiles. However, future studies are warranted to unveil the underlying mechanistic basis of these perturbations. In the present study, triclosan exposure is linked to the microbiome alterations in terms of gene abundance, leading to metagenome change thereby potentially affecting microbiome-related metabolic functions via gene expression. However, the modification of bacterial metabolism can also be induced without altering gene abundance. To profile metabolic capacities in bacteria using metagenomics stands in the initial phase with the absence of corresponding examination of messenger RNA, protein and metabolite [17]. This gap can be filled by more complementary ‘omics’ approaches such as metatranscriptomics, proteomics and metabolomics. Meanwhile, it is of importance to investigate the influence of triclosan on the gut microbiome during specific time windows of susceptibility. Early-life exposure of antibiotics has been associated with an altered microbiome as well as perturbed metabolic functions in mice [52]. In addition, the triclosan exposure for human is lower than the dose that we used in this study, but human exposure goes with much longer periods, therefore there is a need for further study on the dose- and time- dependent effects of triclosan on human gut microbiome.

Several limitations are associated with this study. We performed a 13-week exposure using a single high dose of triclosan, while human exposure is frequently long term and at lower concentrations. Our ongoing study using multiple human-relevant doses aims at better understanding the effect of triclosan on the gut microbiome and host. In addition, the major goal of this study was to define the functional impact of triclosan on the gut microbiome. We have demonstrated that triclosan perturbed the gut microbiome and its functional metagenome. Meanwhile, host response arising from triclosan exposure may also be involved in shaping the gut microbiome. Further characterization of the role of triclosan-induced tissue response in mediating the gut microbiome would provide novel and important insights into triclosan-host-microbiome interaction.

## Conclusions

We combined 16S rRNA gene sequencing and shotgun metagenomics sequencing to probe the effects of triclosan on the mouse gut microbiota and its metabolic functions. Both the compositional and functional changes substantiated the triclosan-induced perturbations of the mouse gut microbiome. A less resilient microbial community with enriched antibiotic and heavy metal resistomes was identified after 13-week triclosan exposure. Our study may provide novel insights regarding the toxicity of triclosan, that is, triclosan could adversely affect host health via microbiome-related perturbations.

## Abbreviations

ENR: Enoyl-[acyl carrier protein] reductase
FMN: Flavin mononucleotide
LPS: Lipopolysaccharide
PCoA: Principle coordinate analysis
UPGMA: Unweighted pair group method with arithmetic mean
